# Reliability of NI-RADS criteria in the interpretation of contrast-enhanced magnetic resonance imaging considering the potential role of diffusion-weighted imaging

**DOI:** 10.1007/s00330-021-07693-4

**Published:** 2021-02-03

**Authors:** Fabian Henry Jürgen Elsholtz, Christoph Erxleben, Hans-Christian Bauknecht, Patrick Dinkelborg, Kilian Kreutzer, Bernd Hamm, Stefan Markus Niehues

**Affiliations:** 1 Department of Radiology, Campus Benjamin Franklin, Charité- Universitätsmedizin Berlin, corporate member of Freie Universität Berlin, Humboldt-Universität zu Berlin, and Berlin Institute of Health, Hindenburgdamm 30, 12203 Berlin, Germany; 2 Department of Neuroradiology, Campus Virchow Klinikum, Charité - Universitätsmedizin Berlin, corporate member of Freie Universität Berlin, Humboldt-Universität zu Berlin, and Berlin and Institute of Health, Augustenburger Platz 1, 13353 Berlin, Germany; 3 Department of Oral and Maxillofacial Surgery, Campus Benjamin Franklin, Charité - Universitätsmedizin Berlin, corporate member of Freie Universität Berlin, Humboldt-Universität zu Berlin, and Berlin Institute of Health, Hindenburgdamm 30, 12203 Berlin, Germany

**Keywords:** Head and neck cancer, Magnetic resonance imaging, Surveillance, Reliability of results

## Abstract

**Objectives:**

To assess inter- and intrareader agreement of the Neck Imaging Reporting and Data System (NI-RADS) used in contrast-enhanced magnetic resonance imaging (MRI) including analysis of diffusion-weighted imaging (DWI), which is currently not part of the NI-RADS criteria.

**Methods:**

This retrospective study included anonymized surveillance contrast-enhanced MRI datasets of 104 patients treated for different head and neck cancers. Three radiologists experienced in head and neck imaging reported findings for the primary site and the neck using NI-RADS criteria in a first step and evaluated DWI sequences for the primary site in a second step. Thirty randomly selected imaging datasets were again presented to the readers. Kappa statistics and observed agreement (A_o_) were calculated.

**Results:**

Interreader agreement across all MRI datasets was moderate (κ_Fleiss_ = 0.53) for NI-RADS categories assigned to the primary site, substantial for NI-RADS categories of the neck (κ_Fleiss_ = 0.67), and almost perfect for DWI of the primary site (κ_Fleiss_ = 0.83). Interreader agreement for the primary site was particularly low in cases of cancer recurrence (κ_Fleiss_ = 0.35) and when categories 2a, 2b, and 3 were combined (κ_Fleiss_ = 0.30). Intrareader agreement was considerably lower for NI-RADS categories of the primary site (range A_o_ = 53.3–70.0%) than for NI-RADS categories of the neck (range A_o_ = 83.3–90.0%) and DWI of the primary site (range A_o_ = 93.3–100.0%).

**Conclusion:**

Interreader agreement of NI-RADS for reporting contrast-enhanced MRI findings is acceptable for the neck but limited for the primary site. Here, DWI has the potential to serve as a reliable additional criterion.

**Key Points:**

*• NI-RADS was originally designed for contrast-enhanced computed tomography with or without positron emission tomography but can also be used for contrast-enhanced magnetic resonance imaging alone.*

*• Overall interreader agreement was acceptable for NI-RADS categories assigned to the neck but should be improved for the primary site, where it was inferior to DWI; similar tendencies were found for intrareader agreement.*

*• DWI is currently no criterion of NI-RADS, but has shown potential to improve its reliability, especially for categories 2a, 2b, and 3 of the primary site.*

**Supplementary Information:**

The online version contains supplementary material available at 10.1007/s00330-021-07693-4.

## Introduction

In the 2018 worldwide cancer statistics, head and neck (HN) cancer including malignancies of the thyroid gland accounted for 8% (1,454,892 patients) of all new cases and 5.2% (494,378) of all deaths [1]. The term HN cancer denotes a heterogeneous group of entities, among which squamous cell carcinoma (SCC) is the largest subgroup [2]. After definitive treatment of HN cancer including surgery, radiotherapy, and chemotherapy, patients are usually put on surveillance imaging programs including contrast-enhanced computed tomography (CECT) or magnetic resonance imaging (CEMRI), possibly combined with positron emission tomography (PET-CT and PET-MRI). Currently, non-standardized reports are common practice. While such reports allow radiologists to individually weight their findings, they can also cause problems [3]. The already complicated head and neck anatomy is even more difficult to interpret in posttreatment situations [4]; therefore, findings, conclusions, and recommendations in radiological reports tend to be heterogeneous, which might reduce their acceptance among surgeons. In 2014, the American College of Radiology (ACR) called upon radiologists to become “patient-centric, data-driven, and outcomes-based” and published the first version of a lexicon and standardized reporting system in 2016 calling it “Neck Imaging Reporting and Data System” (NI-RADS) [5, 6]. NI-RADS thus joins a number of RADS already implemented in clinical routine, which started with the release of the by now well-established BI-RADS in 1997 [7, 8]. NI-RADS reports provide two separate numerical categories between 1 and 4 that stratify the risk of HN cancer recurrence for the primary site and the neck (i.e., cervical lymph nodes) [9]. NI-RADS also addresses the ACR’s imaging 3.0 strategy, which seeks to include clinical decision support directly from radiological reports by linking each category with a concrete recommendation for further patient handling such as shortening the interval until the next imaging examination or performing a biopsy [10]. In this way, NI-RADS standardizes the entire surveillance program. While originally designed for CECT with or without PET, NI-RADS—according to its authors—can also be applied to MRI [10]. Diffusion-weighted imaging, which has already established itself as a standard sequence in head and neck imaging, is not yet a criterion in NI-RADS [11].

The objective of our study was to assess the inter- and intrareader agreement of NI-RADS used in the interpretation of surveillance CEMRI after definitive treatment of head and neck cancer and to gain first insights into the potential of simultaneously acquired DWI data, which are currently not part of the NI-RADS criteria.

## Materials and methods

### Study design and patient population

Our institutional review board approved this retrospective study, and written informed consent for use of their data for scientific purposes was obtained from all patients. We searched our department’s database for suitable patients from all three sites of our hospital using the following inclusion criteria: (1) consecutive patients with available CEMRI datasets of the neck acquired for surveillance after curative treatment of HN cancer; (2) CEMRI datasets including (a) axial contrast-enhanced and fat-suppressed T1-weighted (T1w CE FS) sequences, (b) coronal and fat-suppressed T2-weighted (T2w FS) sequences, (c) axial diffusion-weighted images with corresponding apparent diffusion coefficient (ADC) maps; (3) pretreatment or prior surveillance imaging datasets (CEMRI or CECT) available; (4) availability of reports on either subsequent histopathology or subsequent surveillance imaging no earlier than 6 months later. Datasets with typical MRI artifacts were deliberately not excluded but part of the reporting process as described in the “Image interpretation” paragraph.

The CEMRI datasets included were generated on eight different MRI scanners. Scanner details as well as specific acquisition parameters of the sequences included are presented in Tables 1, 2, 3, and 4 of Electronic Supplementary Material.

### Image interpretation

Three radiologists (A: 15 years; B: 7 years; and C: 6 years of experience in head and neck imaging) contributed reports to this study. Readers A and C are members of the institutional weekly interdisciplinary head and neck tumor board team. The NI-RADS system was well understood as each reader had gained prior experience from interpretation of over 200 CECT and CEMRI datasets using NI-RADS.

All CEMRI datasets included were anonymized and scrubbed of all identifying information and then randomly reordered by using a Mersenne Twister pseudorandom number generator embedded in Microsoft Excel (Version 16.16.10, Microsoft Corporation). The resulting CEMRI datasets were presented on a radiology workstation with two diagnostic monitors and an administrative monitor using Visage Client (Version 7.1.11, Visage Imaging Inc.). The left diagnostic monitor was used to show the current CEMRI dataset, while an earlier imaging dataset (obtained before or after treatment) was presented on the right monitor for comparison. Not until after finishing a NI-RADS report by accessing the axial T1w CE FS and coronal T2w FS sequences intended were readers allowed to view DWI sequences. Readers were provided with a short medical report containing information on the cancer entity, its localization (primary site), and the type of curative therapy (resection, radiation therapy, chemotherapy) to simulate a clinical reporting situation.

For each imaging study interpreted using NI-RADS, two categories are assigned—one for the primary site and another for the neck [9]. Categories range from 1 to 4 and reflect rising probabilities of cancer recurrence. A category of 0 is assigned only if a new baseline study is being interpreted and a prior imaging study exists but is not available at the time of reading. Therefore, a category of 0 could not be assigned in our study setting. A NI-RADS 1 category for the primary site should be assigned when expected posttreatment changes such as diffuse submucosal edema or superficial linear enhancement and non-mass-like distortion are present. NI-RADS 1 for the neck indicates absence of residual abnormal, new or enlarging lymph nodes. NI-RADS 2 for the primary site is divided into 2a for focal mucosal but non-mass-like or nodular enhancement and 2b for deep, ill-defined but not discrete enhancement. NI-RADS 2 for the neck stands for new or enlarging lymph nodes without morphologically abnormal features (central necrosis or extranodular extension). NI-RADS 3 for the primary site is assigned for new or enlarging discrete nodules or masses with intense enhancement. NI-RADS 3 for the neck indicates presence of new or enlarging lymph nodes with central necrosis or extranodular extension. NI-RADS 4 represents clinically or radiologically definite cancer recurrence either at the primary site or in the neck.

Additionally, readers were asked to evaluate the DWI sequences for the primary site by choosing one of two possible options: clear diffusion restriction (positive) and no or ambiguous diffusion restriction (negative). In addition to NI-RADS categories for the primary site and the neck and DWI evaluation for the primary site, readers were asked to also rate image quality for both target sites and for the DWI sequences on a 4-point scale (1 = excellent, 2 = good, 3 = sufficient, 4 = insufficient). More specifically, readers should assign a score of 4 for insufficient image quality when adequate interpretation of the primary site, the neck, or both was prevented by the presence of artifacts.

Three months after completion of retrospective reading of the datasets, readers were asked again to provide NI-RADS categories and evaluate DWI sequences following the same scheme for a subset of 30 randomly selected CEMRI datasets (cases in which an image quality category of 4 was assigned by at least one reader in the first session were excluded). The second session was intended to calculate intrareader agreement.

### Data analysis

**Fig. 1:**
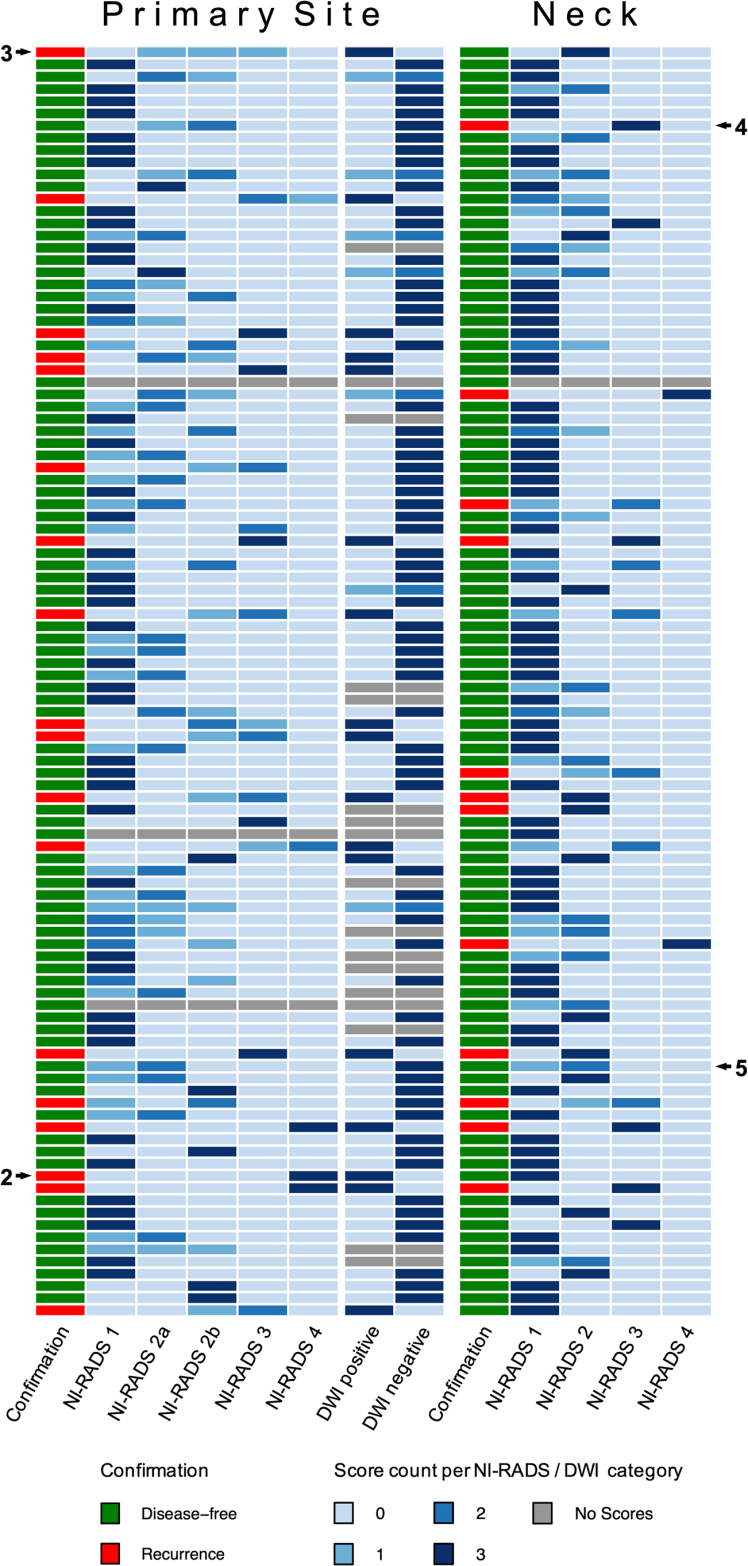
Category distribution chart for all 104 patients. Category counts are coded as shades of blue. The results of the confirmation studies are coded in green and red. Numbers to the left and to the right side indicate figures providing examples for illustration

“RStudio” (Version 1.1.383, RStudio) was used both for statistical data analysis (“irr” package) and for creating the heatmap (Fig. 1, “gplots” package).

Fleiss’ kappa (κ_Fleiss_) and Conger’s kappa (κ_Conger_) were calculated to test interreader agreement between all three readers. Intrareader agreement was tested by calculation of Cohen’s kappa (κ_Cohen_). Additionally, the observed agreement (A_O_, i.e., all three readers assigned the same category) was calculated.

κ_Fleiss_, κ_Conger_, and κ_Cohen_ were interpreted as recommended by Landis and Koch: 0.01–0.20 = “slight agreement,” 0.21–0.40 = “fair agreement,” 0.41–0.60 = “moderate agreement,” 0.61–0.80 = “substantial agreement,” and 0.81–1.0 = “(almost) perfect agreement” [12].

Subgroups were formed according to the results of the confirmation study (disease-free versus cancer recurrence) and the NI-RADS categories assigned by the majority of the readers (2a/2b/3 versus 1/4 merged; in case each reader provided a different category for a target, the category assigned by the most experienced reader A was decisive). As a secondary outcome, recurrence rates for all patients and for each NI-RADS category were calculated.

## Results

A total of 104 patients (35 female, 69 male) were finally accepted for retrospective analysis. They had a median age of 60 years with a range of 18–89 years. The median follow-up interval for imaging confirmation studies was 219 days (range 184–450 days). Table 1 provides the distribution of HN cancer entities included in our study along with subsites and treatment plans. Pretreatment or prior surveillance imaging datasets were acquired by CEMRI in 64 cases and CECT in 40 cases.

**Table 1:** Distribution of cancer entities, sites, and treatment plans

Characteristics	Count (%)
Cancer entity	
Squamous cell carcinoma	77 (74.0)
Adenocarcinoma	10 (9.6)
Mucoepidermoid carcinoma	7 (6.7)
Acinic cell carcinoma	2 (1.9)
Adenoid cystic carcinoma	2 (1.9)
Papillary thyroid carcinoma	2 (1.9)
Cystadenocarcinoma	1 (1.0)
Follicular thyroid carcinoma	1 (1.0)
Adenosarcoma	1 (1.0)
Epithelial myoepithelial carcinoma	1 (1.0)
Primary site	
Oral cavity	29 (27.9)
Oropharynx	25 (24.0)
Nasopharynx	9 (8.7)
Hypopharynx	5 (4.8)
Larynx (supraglottic)	6 (5.8)
Larynx (glottic)	4 (3.9)
Parotid gland	19 (18.3)
Submandibular gland	3 (2.9)
Thyroid gland	3 (2.9)
Trachea	1 (1.0)
Treatment	
Surgery only	34 (32.7)
Radiotherapy only	4 (3.9)
Surgery + radiotherapy	36 (34.6)
Surgery + radiochemotherapy	29 (27.9)

**Fig. 2:**
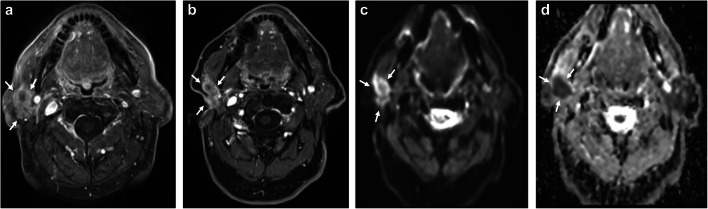
**a**-**d** Example case illustrating high agreement for the primary site. Axial T1w CE FS images obtained before (**a**) and 6 months after total parotidectomy and adjuvant radiochemotherapy (**b**) in a patient diagnosed with adenocarcinoma located in the right parotid gland (white arrows). Image **b** again shows nodular enhancement at the primary site (white arrows), for which all three readers assigned a category of 4. All three readers also identified corresponding diffusion restriction in DWI/ADC maps (**c** and **d**, white arrows). Histopathology confirmed cancer recurrence

**Fig. 3:**
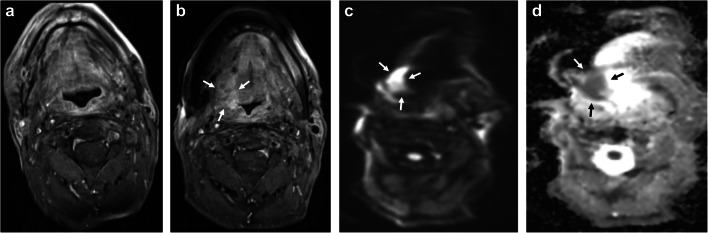
**a**-**d** Example case illustrating low agreement for the primary site. Axial T1w CE FS images obtained 12 (**a**) and 24 months (**b**) after definitive radiochemotherapy in a patient diagnosed with of SCC located in the right root of the tongue. Image **b** shows new enhancement at the primary site (white arrows), for which readers assigned categories of 2a, 2b, and 3. All three readers identified corresponding diffusion restriction in DWI/ADC maps despite severe geometric distortion (**c** and **d**, white arrows). Histopathology revealed cancer recurrence

**Fig. 4:**
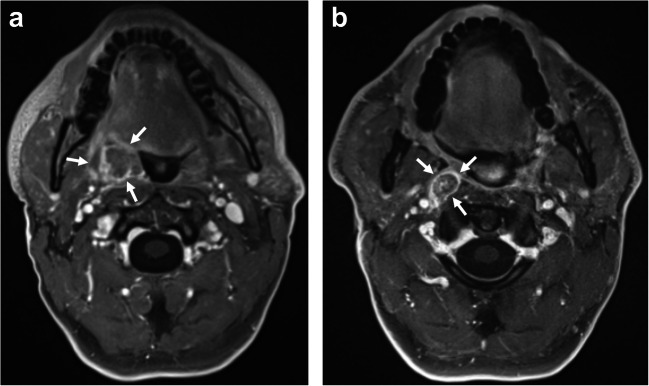
**a**, **b** Example case illustrating high agreement for the neck. Axial T1w CE FS images obtained before (**a**) and 6 months (**b**) after surgical resection in a patient diagnosed with SCC located in the right palatine tonsil (white arrows in **a**). Posttreatment image **b** shows a new right retropharyngeal lymph node (white arrows), for which all three readers assigned a category of 3, 3, and 3. Histopathology confirmed cancer recurrence

**Fig. 5:**
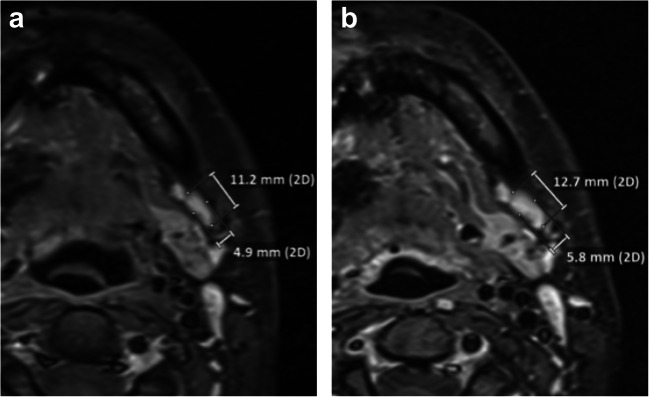
**a**, **b** Example case illustrating low agreement for the neck. Magnified section of axial T1w CE FS images obtained 6 (**a**) and 12 months (**b**) after lateral parotidectomy and radiotherapy in a patient diagnosed with adenoid cystic carcinoma located in the right parotid gland. Image **b** shows a slightly enlarged lymph node at level Ib on the left side, for which readers assigned categories of 1, 2, and 2. The subsequent MRI obtained 6 months later showed no further increase in size

With regard to image quality, NI-RADS categories of 101 datasets for the primary site (3 ratings of 4 = insufficient) and 103 datasets for the neck (1 rating of 4 = insufficient) as well as evaluations of 87 datasets for DWI (17 ratings of 4 = insufficient) were accepted for further analysis. Rating distributions for all 104 patients as well as the results of the confirmation studies are presented in a heatmap in Fig. 1. Examples illustrating high and low interreader agreement for NI-RADS categories assigned to the primary site and the neck are shown in Figs. 2, 3, 4, and 5.

The results for interreader agreement are compiled in Table 2. Interreader agreement was moderate for NI-RADS categories of the primary site (κ_Fleiss_ = 0.53, κ_Conger_ = 0.54, A_o_ = 55.5%), substantial for NI-RADS categories of the neck (κ_Fleiss_ = 0.67, κ_Conger_ = 0.68, A_o_ = 75.7%), and almost perfect for evaluation of DWI of the primary site (κ_Fleiss_ = 0.83, κ_Conger_ = 0.83, A_o_ = 92.0%). Regarding assignment of NI-RADS categories to the primary site, interreader agreement was particularly low in cases of cancer recurrence (κ_Fleiss_ = 0.35, κ_Conger_ = 0.38, A_o_ = 38.9%) and when categories 2a, 2b, and 3 were combined (κ_Fleiss_ = 0.30, κ_Conger_ = 0.34, A_o_ = 24.5). Conversely, interreader agreement for DWI was perfect (κ_Fleiss_ = 1.00, κ_Conger_ = 1.00, A_o_ = 100.0%) or almost perfect (κ_Fleiss_ = 0.83, κ_Conger_ = 0.83, A_o_ = 89.1%) in those subgroups.

**Table 2:** Interreader agreement for all three readers. κ_Fleiss_ = Fleiss’ kappa, κ_Conger_ = Conger’s kappa, A_o_ = observed agreement (in %)

Group	Primary site						Neck		
	NI-RADS			DWI			NI-RADS		
	κ_Fleiss_	κ_Conger_	A_o_	κ_Fleiss_	κ_Conger_	A_o_	κ_Fleiss_	κ_Conger_	A_o_
Overall	0.53	0.54	55.5	0.83	0.83	92.0	0.67	0.68	75.7
Disease-free	0.47	0.49	59.0	0.23	0.24	87.3	0.59	0.59	75.8
Cancer recurrence	0.35	0.38	38.9	1.00	1.00	100.0	0.74	0.74	75.0
Categories 2a, 2b, and 3 merged	0.30	0.34	24.5	0.83	0.83	89.1	N/A	N/A	N/A
Categories 1 and 4 merged	0.53	0.54	84.6	0.84	0.84	95.1	N/A	N/A	N/A

Results for intrareader agreement determined in 30 cases are shown in Table 3. Overall intrareader agreement was considerably lower for NI-RADS categories of the primary site (range κ_Cohen_ = 0.76–0.84, range A_o_ = 53.3–70.0%) than for NI-RADS categories of the neck (range κ_Cohen_ = 0.85–0.90, range A_o_ = 83.3–90.0%) and DWI of the primary site (range κ_Cohen_ = 0.63–1.00, range A_o_ = 93.3–100.0%).

**Table 3:** Intrareader agreement for all three readers. κ_Cohen_ = Cohen’s kappa, A_o_ = observed agreement (in %)

Reader	Primary site				Neck	
	NI-RADS		DWI		NI-RADS	
	κ_Cohen_	A_o_	κ_Cohen_	A_o_	κ_Cohen_	A_o_
A	0.76	53.3	0.84	96.7	0.85	83.3
B	0.84	70.0	1.00	100.0	0.86	83.3
C	0.77	53.3	0.63	93.3	0.90	90.0

Table 4 summarizes recurrence rates for all datasets and for the individual NI-RADS categories calculated from the results of confirmation studies—histopathological examinations or further follow-up imaging.

**Table 4:** Category counts, recurrence counts, and recurrence rates by NI-RADS category and total based on majority decisions (the most experienced reader A was decisive in case of tied category counts). For comparison, the last two columns list the recurrence rates reported in the two previously published studies investigated the validity of NI-RADS [19, 22]

NI-RADS category	Category counts	Recurrence counts	Recurrence rates		
			Study	Krieger et al	Dinkelborg et al
Primary 1	48	0	0%	3.5%	1.0%
Primary 2a	22	1	4.6%	18.4%	7.1%
Primary 2b	15	3	20.0%		5.6%
Primary 3	12	10	83.3%	54.6%	66.7%
Primary 4	4	4	100.0%	N/A	100.0%
Primary total	101	18	17.8%	8.9%	7.6%
Neck 1	66	0	0%	4.0%	0.5%
Neck 2	23	3	13.0%	15.0%	7.0%
Neck 3	12	7	58.3%	70.0%	80.0%
Neck 4	2	2	100.0%	N/A	100.0%
Neck total	103	12	11.7%	7.9%	6.2%

## Discussion

This study was conducted to obtain first results on the reliability of NI-RADS in the interpretation of CEMRI including DWI, which is currently no criterion of NI-RADS. Overall, our results show acceptable interreader agreement for NI-RADS categories assigned to the neck, while further improvement is desirable for primary cancer sites. In particular, interreader agreement was low in recurrent cancer and for the combination of categories 2a, 2b, and 3. Overall intrareader agreement showed similar tendencies but was acceptable even for NI-RADS categories assigned to the primary site. For DWI, we found excellent interreader agreement.

So far, a single other study on the reliability of NI-RADS has been published; however, this study investigated surveillance CECT datasets after treatment of oral and oropharyngeal SCC, and categories were assigned by four instead of three readers [13]. While not directly comparable with our design, this study found moderate overall interreader agreement for both the primary site (κ_Fleiss_ = 0.48) and the neck (κ_Fleiss_ = 0.50). Intrareader agreement was almost perfect (κ_Cohen_ = 0.85–0.96) for both the primary site and the neck (0.89–0.95). A major difference to our MRI study is that agreement for NI-RADS categories of the primary site was higher in patients with confirmed cancer recurrence than patients without recurrence. This discrepancy could be due to a higher proportion of less clear cases in our MRI study or reader-dependent differences in the interpretation of abnormal findings. Finally, compared with the CECT study, our patient population better reflects the claim of NI-RADS to be applicable to any HN cancer.

Since experience with NI-RADS is still limited, comparison with published data on the reliability of other RADS is worthwhile. Two recent studies are of particular interest here because they arrived at very different results. For discussion and comparison of the overall agreements reported by these studies and quoted below, we use the same interpretations of the statistical parameters as defined by us, though they were not used by the authors of these studies. The first study investigated the current version of BI-RADS and found moderate interreader agreement (κ_Fleiss_ = 0.57) while intrareader agreement was substantial or almost perfect (range of κ_Cohen_ = 0.72–0.81) [14]. In contrast, the second study, which investigated the current version of PI-RADS, showed fair interreader agreement (κ_Fleiss_ = 0.24) and a moderate intrareader agreement range of Cohen’s kappa of 0.43–0.67 [15].

We measured inter- and intrareader agreement using two approaches—calculation of observed agreement (the proportion of perfect agreements between readers) and calculation of the widely used kappa statistics (which takes agreement expected by chance into account). Kappa statistics are suitable for binary (DWI) and categorical or ordinal data. Fleiss and Congers kappa, unlike Cohen’s kappa, can be used to calculate agreement between more than two readers. For interpretation of DWI results in cases confirmed as disease-free, the kappa statistic is very low (κ_Fleiss_ = 0.23) and does not match the observed agreement (A_o_ = 87.3%). This paradoxical situation can occur when most of the subjects examined are assigned to the same category [16, 17]. In our study, we observed this paradox in the DWI disease-free subgroup, where the three readers combined reported a diffusion restriction (positive) in 11 instances and no restriction (negative) in 199 instances. This proportion was much more balanced across all patients who underwent DWI with 59 positive versus 202 negative instances.

Another issue to be addressed is poor image quality. In our study, image quality was considered insufficient for three datasets for the primary site and one dataset for the neck. To report such cases in a standardized manner as well, the existing categories might be supplemented by a category for non-diagnostic image quality (e.g., NI-RADS N/A).

In discussions with the readers after the reading sessions, two issues emerged. First, handling of the NI-RADS 2 category for the neck was unclear in some cases with measurable increases in size of unclear relevance. Figure 5 illustrates such a case, in which a very subtle enlargement of a contralateral lymph node (not a typical draining lymph node) is apparent. According to the RECIST criteria, only a short axis diameter larger than 10 mm would be considered relevant [18]. This problem was already noted in the above-quoted study investigating NI-RADS reliability in CECT [13]. We think that a statement regarding this issue would be a valuable supplement to the NI-RADS criteria. The second issue is that one reader reconsidered the NI-RADS category assigned in light of the recommended clinical management that would automatically follow from the category. While this might indicate a potential bias in this reader, it is exactly what is likely to happen in clinical practice [19].

The full spectrum of NI-RADS criteria can only be used with PET, which is likely to result in higher interreader agreement for the primary site. However, given the high interreader agreement we found for the evaluation of DWI, its integration into NI-RADS could have the same effect as additional consideration of PET findings. This is of interest in light of the high cost of PET [20, 21]. Differentiation between categories 2b and 3 would probably benefit most from additional DWI. An example illustrating this situation is given in Fig. 3; in this case, DWI was highly suspicious for primary cancer recurrence. The readers in our study unanimously agree that, with its higher soft tissue contrast, CEMRI might be particularly susceptible to discrepant interpretation in categories 2a, 2b, and 3. Another possible reason for disagreement is that there is an inherent problem in differentiating category 3 and 4, both for the primary site and the neck: while category 3 has clearly defined criteria, category 4 leaves room for interpretation. The recurrence rates calculated as secondary outcome in our study support the high discriminatory power of NI-RADS categories already demonstrated in previous studies [19, 22] with one notable exception. For patients assigned category 3 for the neck, these two studies report higher recurrence rates of 70.0% and 80.0% compared with the rate of 58.3% in our study. Possible explanations are the small sample size for the validity check and an overinterpretation of the “abnormal features.” If these trends are confirmed, a feature-specific investigation could provide additional insights.

Our study has several limitations. MRI datasets acquired on eight different MRI scanners meeting the inclusion criteria were analyzed. Even if this realistically reflects the clinical setting, where radiologists also have to deal with images acquired on different machines, a potential bias remains because consistent interpretation of different image impressions by the readers cannot be taken for granted. A coronal T2-weighted FS sequence is standard in many MRI examinations of the neck and was therefore included in our analysis, although it is not specifically mentioned in the NI-RADS criteria. In this respect, the methodology used in our single-center study may differ from approaches used by other investigators in figure studies. The subgroup of non-SCC entities was very heterogeneous in our study, as was the distribution of primary cancer sites. A subanalysis was therefore not reasonable, but could provide important results in future adequately powered for such an analysis.

In conclusion, the results of our study suggest that NI-RADS categories can be statistically reliable when used in the interpretation of CEMRI. While interreader agreement for NI-RADS categories of the neck is acceptable, there is still need for improving assessment of the primary site, especially for NI-RADS categories of 2a, 2b, and 3. This limitation could be overcome by supplementary DWI, for which we found high interreader agreement.

## Supplementary Information

ESM 1 (DOCX 20 kb)

## Abbreviations

ADC: Apparent diffusion coefficient
CE: Contrast-enhanced
DWI: Diffusion-weighted imaging
FS: Fat-suppressed
HN: Head and neck
NI-RADS: Neck Imaging Reporting and Data System
SCC: Squamous cell carcinoma
T1w: T1-weighted
T2w: T2-weighted

## Declarations

### Guarantor

The scientific guarantor of this publication is Fabian Henry Jürgen Elsholtz.

### Statistics and biometry

No complex statistical methods were necessary for this paper.

### Methodology

• retrospective

• diagnostic or prognostic study

• performed at one institution
