# Supplementary material for: Reliability of NI-RADS criteria in the interpretation of contrast-enhanced magnetic resonance imaging considering the potential role of diffusion-weighted imaging
Source: Eur Radiol. 2021 Feb 3;31(8):6295–304. doi: 10.1007/s00330-021-07693-4 (PMC8270833; doi:10.1007/s00330-021-07693-4)
Supplement: Supplementary file 1 — (DOCX 20 kb) [file 330_2021_7693_MOESM1_ESM.docx]

**Electronic Supplementary Material**

| **#** | **Manufacturer** | **Model** | **Field strength** |
| --- | --- | --- | --- |
| 1 | Siemens | Aera | 1.5 T |
| 2 | Siemens | Skyra | 3 T |
| 3 | Siemens | Vida | 3 T |
| 4 | Siemens | Skyra | 3 T |
| 5 | Siemens | Avanto | 1.5 T |
| 6 | Siemens | Aera | 1.5 T |
| 7 | Siemens | Skyra | 3 T |
| 8 | Siemens | Biograph | 3 T |

**Table 1.** MRI scanner details. Abbreviation: T = Tesla.

| **#** | **Type** | **TR** | **TE** | **FLIP** | **Slice thickness** | **Matrix** |
| --- | --- | --- | --- | --- | --- | --- |
| 1 | TSE FS | 630 | 11 | 160 | 5 | 384 x 269 |
| 2 | TSE FS | 850 | 12 | 160 | 4 | 320 x 288 |
| 3 | VIBE DIXON | 5.6 | 2.5 | 12 | 3 | 288 x 295 |
| 4 | STARVIBE FS | 4.0 | 1.9 | 9 | 1 | 288 x 288 |
| 5 | VIBE FS | 16 | 7.1 | 10 | 2 | 384 x 278 |
| 6 | STARVIBE FS | 4.1 | 2.2 | 10 | 1.3 | 320 x 320 |
| 7 | STARVIBE FS | 4.03 | 1.97 | 9 | 1 | 320 x 320 |
| 8 | STARVIBE FS | 9.46 | 2.06 | 12 | 1 | 320 x 320 |

**Table 2.** Specific parameters of the axial T1-weighted contrast-enhanced fat-saturated sequences. Slice thickness is provided in mm, matrix is provided in pixels. Abbreviations: TR = repetition time (in ms), TE = echo time (in ms), Flip = flip angle (in degrees of arc), TSE = turbo spin echo, FS = fat-saturated, VIBE: volumetric interpolated breath-hold examination.

| **#** | **Type** | **TR** | **TE** | **FLIP** | **ST** | **MAT** |
| --- | --- | --- | --- | --- | --- | --- |
| 1 | TIRM | 5470 | 65 | 150 | 3 | 320 x 224 |
| 2 | TSE DIXON | 4200 | 89 | 126 | 3 | 320 x 288 |
| 3 | TIRM | 3140 | 37 | 158 | 4 | 320 x 224 |
| 4 | TIRM | 3350 | 37 | 127 | 3 | 320 x 224 |
| 5 | TIRM | 4600 | 81 | 150 | 4 | 256 x 179 |
| 6 | TIRM | 3300 | 35 | 160 | 3 | 320 x 224 |
| 7 | TSE DIXON | 8960 | 81 | 164 | 3 | 320 x 256 |
| 8 | TIRM | 3300 | 37 | 160 | 3 | 320 x 224 |

**Table 3.** Specific parameters of the coronal T2-weighted contrast-enhanced fat-saturated sequences. Abbreviations: TR = repetition time (in ms), TE = echo time (in ms), Flip = flip angle (in degrees of arc), ST = slice thickness (in mm), MAT = matrix (in pixels), TSE = turbo spin echo, TIRM = turbo inversion recovery.

| **#** | **Type** | **TR** | **TE** | **FLIP** | **ST** | **MAT** | **b** |
| --- | --- | --- | --- | --- | --- | --- | --- |
| 1 | EPI | 7812 | 75 | 90 | 5 | 128 x 88 | 800 |
| 2 | EPI | 7788 | 55 | 90 | 5 | 128 x 88 | 800 |
| 3 | EPI | 10200 | 48 | 90 | 3 | 128 x 88 | 800 |
| 4 | EPI | 20000 | 48 | 90 | 4 | 128 x 88 | 800 |
| 5 | EPI | 3900 | 88 | 90 | 5 | 128 x 128 | 800 |
| 6 | RESOLVE | 9210 | 116 | 180 | 3 | 176 x 176 | 800 |
| 7 | RESOLVE | 4510 | 63 | 180 | 3.5 | 180 x 118 | 800 |
| 8 | RESOLVE | 3550 | 63 | 180 | 3 | 200 x 117 | 800 |

**Table 4.** Specific parameters of the axial DWI sequences. Abbreviations: TR = repetition time (in ms), TE = echo time (in ms), Flip = flip angle (in degrees of arc), ST = slice thickness (in mm), MAT = matrix (in pixels), b = b-value (in s/mm^2^), EPI = echo-planar imaging, RESOLVE = readout segmentation of long variable echo trains).
